# The functional comorbidity index had high inter-rater reliability in patients with acute lung injury

**DOI:** 10.1186/1471-2253-12-21

**Published:** 2012-09-13

**Authors:** Eddy Fan, Jeneen M Gifford, Satish Chandolu, Elizabeth Colantuoni, Peter J Pronovost, Dale M Needham

**Affiliations:** 1 Division of Pulmonary and Critical Care Medicine, Johns Hopkins University, Baltimore, MD, USA; 2 Interdepartmental Division of Critical Care Medicine, University of Toronto, Toronto, Canada; 3 Department of Critical Care Medicine, National Institutes of Health, Bethesda, MD, USA; 4 Division of Pulmonary and Critical Care Medicine, Johns Hopkins University, Baltimore, MD, USA; 5 Department of Biostatistics, Bloomberg School of Public Health, Johns Hopkins University, Baltimore, MD, USA; 6 Department of Anesthesiology and Critical Care Medicine, Johns Hopkins University, Baltimore, MD, USA; 7 Division of Pulmonary and Critical Care Medicine, and Department of Physical Medicine and Rehabilitation, Johns Hopkins University, Baltimore, MD, USA; 8 Mount Sinai Hospital, 600 University Avenue, Room 18-232, Toronto, ON, M5G 1X5, Canada

**Keywords:** Comorbidity, Intensive care unit, Reproducibility of results, Respiratory distress syndrome, Adult

## Abstract

**Background:**

The Functional Comorbidity Index (FCI) was recently developed to predict physical function in acute lung injury patients using comorbidity data. Our objectives were to determine: (1) the inter-rater reliability of the FCI collected using in-patient discharge summaries (primary objective); and (2) the accuracy and predictive validity of the FCI collected using hospital discharge summaries and admission records versus complete chart review (secondary objectives).

**Methods:**

For reliability, we evaluated the FCI’s intraclass correlation coefficient (ICC) among trained research staff performing data collection for 421 acute lung injury patients enrolled in a prospective cohort study. For validity and accuracy, we compared the detection of FCI comorbidities across three types of inpatient medical records, and the association of the respective FCI scores obtained with patients’ SF-36 physical function subscale (PFS) scores at 1-year follow-up.

**Results:**

Inter-rater reliability was near-perfect (ICC 0.91; 95% CI 0.89-0.94). Hospital admission records and discharge summaries (vs. complete chart review) significantly underestimated the total FCI score. However, using multivariable linear regression, FCI scores collected using each of the three types of inpatient medical records had similar associations with PFS, suggesting similar predictive value.

**Conclusions:**

Data collection using in-patient discharge summaries represents a reliable and valid method for collecting FCI comorbidity information.

## Background

Survivors of critical illness have significant impairments in physical function after hospital discharge [1-4]. However, there is a complex relationship between survivors’ long-term outcomes and their pre-admission comorbidities, critical illness and ICU management. Adjustment for patients’ baseline comorbidities is essential to understand the independent contributions of critical illness and ICU management to survivors’ long-term outcomes. This issue may be especially important in the elderly, in whom many chronic illnesses often coexist [5-7].

Comorbidity information can be obtained from a variety of sources, including medical records, patient self-report, and large administrative databases. Clinical studies of hospitalized patients commonly rely on medical records to measure comorbid diseases [8]. However, review of medical records is time consuming, particularly if the entire medical record is reviewed during a lengthy and complex hospitalization, which is common with critically ill patients. Rather than reviewing the entire medical record, researchers may collect comorbidity data using two alternative methods: (1) prospective collection from hospital admission records available at the time of patient enrollment, or (2) retrospective collection based on patients’ hospital discharge summary. The latter option is particularly attractive because these discharge summaries are frequently available electronically avoiding the need for review of paper-based medical charts. For these two data collection methods to be useful for clinical research, they must introduce minimal bias in the measurement of comorbidity.

The Functional Comorbidity Index (FCI) was recently developed to predict physical function based on comorbidity data [9] and was validated in critically ill patients with acute lung injury (ALI) [10]. With the FCI, relatively little is known about its inter-rater reliability, and the validity of these 3 different methods of data collection (e.g., prospective collection from admission records versus retrospective collection from discharge summary versus complete medical chart review). Consequently, using a cohort of critically ill patients with ALI, our objectives were to: (1) determine the inter-rater reliability of the FCI collected using hospital discharge summary records; and (2) compare the accuracy and predictive validity of two abbreviated data collection methods (i.e., retrospective collection from hospital discharge summaries and prospective collection from hospital admission records) with complete medical record review (gold standard) on the FCI.

## Methods

### Data sources

Data for this research was obtained from the Improving Care of Acute Lung Injury Patients (ICAP) study [11]. The ICAP study is a multi-site prospective cohort study which evaluates long-term outcomes of ALI survivors. The ICAP study was approved by the Institutional Review Board of the Johns Hopkins University and all participating study sites.

### The functional comorbidity index (FCI)

The FCI is a sum of 18 self-reported comorbid conditions with a score of 0 to 18 (Table 1) [9]. Obesity (i.e., BMI > 30) was calculated using height and weight information abstracted from the medical chart and was not assessed directly by any data collection method, leaving 17 comorbid conditions for evaluation in this study. A higher FCI score indicates greater comorbidity and is associated with impairment in physical function 1 year later, as measured by the Medical Outcomes Study Short-Form 36 (SF-36) Physical Function subscale (PFS) [12]. The SF-36 is a validated, 36-item generic health-related quality of life instrument. The PFS of the SF-36 instrument consists of 10 items which assess self-reported ability with walking, climbing stairs, lifting, bathing and dressing.

**Table 1:** **The Functional Comorbidity Index [**[9]**]**

	**Comorbidity**
1	Arthritis (rheumatoid and osteoarthritis)
2	Osteoporosis
3	Asthma
4	Chronic obstructive pulmonary disease (COPD), acute respiratory distress syndrome (ARDS), or emphysema
5	Angina
6	Congestive heart failure (or heart disease)
7	Heart attack (myocardial infarction)
8	Neurological disease (e.g., multiple sclerosis or Parkinson’s disease)
9	Stroke or transient ischemic attack (TIA)
10	Peripheral vascular disease
11	Diabetes types I and II
12	Upper gastrointestinal disease (e.g., ulcer, hernia, reflux)
13	Depression
14	Anxiety or panic disorders
15	Visual impairment (e.g., cataracts, glaucoma, macular degeneration)
16	Hearing impairment (i.e., very hard of hearing, even with hearing aids)
17	Degenerative disc disease (e.g., back disease, spinal stenosis, or severe chronic back pain)
18	Obesity and/or body mass index (BMI) > 30

### Inter-rater reliability of retrospective data collection for FCI

Our primary objective was evaluating the inter-rater reliability of the FCI based on the results of two reviewers (EF and JMG) who performed independent data collection from hospital discharge summaries on a convenience sample of the first 421 patients in the ICAP study cohort. To evaluate the generalizability of this inter-rater reliability finding, three additional independent research assistants similarly performed data collection from hospital discharge summaries on a 30 patient convenience subset of this sample. Comorbidities were defined as any distinct patient diagnosis that existed prior to the index hospitalization for ALI.

### Accuracy and predictive validity of prospective and retrospective data collection for FCI

We calculated the accuracy of FCI’s data collection for the individual comorbidities and the total FCI score based on comparison of (1) prospective data collection from the hospital admission records, and (2) retrospective collection from hospital discharge summaries versus complete medical chart review (gold standard) performed by a single data abstractor (EF).

We compared the predictive validity of the total FCI score, obtained by each of the three data collection methods, by comparing the association between each FCI score and SF-36 PFS score at 1 year. To contrast with these findings regarding the FCI, we also evaluated the predictive validity of the Charlson Comorbidity Index (CCI) [13] and the chronic health points comorbidity measure of the Acute Physiology and Chronic Health Evaluation (APACHE) II score [14] for SF-36 PFS score at 1 year.

### Power and sample size considerations

Using the method of Walter *et al*. [15], an analysis of inter-rater reliability, completed by two reviewers for 421 patients, had >99% power for a target reliability of 0.90, exceeding a minimum reliability (i.e., minimally acceptable level of reliability) of 0.80 with 1-sided α = 0.05.

For comparisons of inter-rater reliability among all five reviewers, a sample size of 28 patients was required for 80% power for an obtained reliability of 0.90, exceeding a minimum reliability of 0.80 with 1-sided α = 0.05. Hence, for the secondary objective, 30 charts (randomly selected from patients enrolled in the ICAP study at the Johns Hopkins Hospital study site) were reviewed by the three additional independent reviewers, as previously described, to ensure at least 80% power for all comparisons.

In comparing prospective and retrospective data collection versus complete chart review (gold standard), a sample size of 59 patients was calculated to detect a 1.5-unit difference in FCI score, with 95% power and a 2-sided α = 0.05 (assuming a standard deviation in FCI score of 1.7 [10]). There is currently no information on a minimum important difference (MID) for the FCI score. Therefore, we extrapolated the estimated MID for FCI from its relationship with the SF-36 PFS. Assuming a standard deviation of 30 [4,16] and an estimated MID of 10 for the SF-36 PFS [17], the MID for PFS corresponds to 0.33 standard deviation units. Using the standardized regression coefficient (−0.361) for the association of FCI and SF-36 PFS [16], a 1.5-unit difference in FCI score corresponds to a MID in the SF-36 PFS.

### Statistical methods

Descriptive statistics are reported as medians (interquartile range [IQR]) or proportions, and compared using the Wilcoxon signed-rank test or McNemar’s test, respectively.

Inter-rater reliability between data collectors was evaluated using an intraclass correlation coefficient (ICC) with 95% confidence intervals (CI) for each FCI individual comorbidity and for the total FCI score. Using the nomenclature of Shrout and Fleiss [18], an ICC (2,1) was calculated to provide a measure of inter-rater reliability that could be generalized beyond the study. The primary comparison was between the 2 reviewers who reviewed the first 421 patients in the ICAP study cohort. For the secondary objective, we compared all 5 reviewers using the smaller patient subset. In addition to reporting ICC quantitatively, the level of agreement from ICC was reported qualitatively according to the classification system of Landis and Koch [19].

For the accuracy analysis (n = 59), the prevalences of individual FCI comorbidities for each of the two data collection methods were reported as proportions and separately compared with the gold standard (complete chart review) using McNemar’s test. Sensitivity, specificity, and positive/negative predictive values using each of the hospital admission records and discharge summaries versus the gold standard was determined for the individual FCI comorbidities.

For predictive validity, the association between FCI score calculated using each of the three different data collection methods and the SF-36 PFS score at 1 year after ALI was determined using ordinary least-squares linear regression with and without adjustment for age and gender. For this analysis, we used a 47-patient subset of the original 59 patients sample size with complete chart review who survived and completed the SF-36 at 1 year. Marked discrepancies in these bivariate and multivariable associations from the linear regression models were detected by visual inspection of the overlap of their 95% confidence intervals [20]. The amount of variance explained in SF-36 PFS score at 1 year, as measured by R^2^ for the multivariable model, was compared between the three different data collection methods. We also evaluated the amount of variance explained in SF-36 PFS score at 1 year with models using CCI and chronic health points comorbidity measure of the APACHE II score instead of FCI.

All statistical analyses were performed using STATA statistical software version 11.2 (Stata Corporation, College Station, TX). A two-sided p-value <0.05 was used to indicate statistical significance.

## Results

### Characteristics of study patients

The median age (IQR) for the cohort (n = 421) was 52 (42–63) years and 44% were female. Median (IQR) severity of illness at ICU admission, as measured by APACHE II score, was 26 (20–33). Median (IQR) Charlson and Functional Comorbidity indices, using data from hospital admission records, was 2 (1–4) and 1 (0–2), respectively. The median (IQR) SF-36 PFS score at 12 months in the 146 patients who survived (out of a possible 156 patients who were available for follow-up at 12 months) and had this assessment done was 55 (25–80).

### Inter-rater reliability of hospital discharge summary for FCI

The inter-rater reliability of the FCI score obtained using hospital discharge summaries (n = 421) between the two original reviewers was near-perfect, with an ICC 0.91 (95% CI 0.89-0.94). Using a subset of FCI scores (n = 30) obtained using hospital discharge summaries by all 5 data collectors, inter-rater reliability remained “substantial”, with ICC (95% CI) for all five data collectors, two original reviewers, and three research assistants: 0.78 (0.66-0.87), 0.89 (0.80-0.95), and 0.71 (0.54-0.84), respectively.

### FCI individual comorbidities and total score by data collection method

The prevalences of individual FCI comorbidities obtained by prospective collection from hospital admission records and retrospective collection from hospital discharge summaries, compared to complete chart review (gold standard) are presented in Table 2. As compared to the gold standard, review of hospital admission records detected 8 of the 17 FCI comorbidities significantly less frequently: arthritis; asthma; COPD, emphysema, or ARDS; congestive heart failure (or heart disease); neurological disease; upper GI disease; depression; and anxiety. Moreover, the median (IQR) FCI score calculated from the hospital admission records was significantly lower than that obtained from the gold standard complete chart review (1 [0–1] vs. 2 [1–3], p < 0.01).

**Table 2:** Prevalence of Individual FCI Comorbidities by Data Collection Method (n = 59)

**FCI Comorbidity, n (%)**	**Gold Standard Chart Abstraction**	**Prospective: Admission Records**	p-value*	**Retrospective: Discharge Summaries**	p-value**
Arthritis (rheumatoid and osteoarthritis)	5 (8)	1 (3)	0.05	3 (5)	0.41
Osteoporosis	0 (0)	0 (0)	n/a	1 (2)	0.32
Asthma	7 (12)	2 (3)	0.03	6 (10)	0.32
COPD, emphysema, or ARDS	7 (12)	0 (0)	<0.01	5 (8)	0.32
Angina	1 (2)	0 (0)	0.32	0 (0)	0.32
Congestive heart failure (or heart disease)	10 (17)	5 (8)	0.05	8 (14)	0.16
Myocardial infarction	2 (3)	2 (3)	1.00	1 (2)	0.32
Neurological disease (e.g., MS or Parkinson’s)	6 (10)	0 (0)	0.01	8 (14)	0.16
Stroke or TIA	4 (7)	5 (8)	0.32	4 (7)	1.00
Peripheral vascular disease	4 (7)	1 (3)	0.08	3 (5)	0.32
Diabetes type I or II	10 (17)	9 (15)	0.56	10 (17)	1.00
Upper GI disease (e.g., ulcer, hernia, reflux)	23 (39)	4 (7)	<0.01	24 (41)	0.71
Depression	15 (25)	3 (5)	<0.01	11 (19)	0.16
Anxiety or panic disorders	6 (10)	0 (0)	0.01	6 (10)	1.00
Visual impairment (e.g., cataracts, glaucoma)	0 (0)	0 (0)	1.00	1 (2)	0.32
Hearing impairment (e.g., hard of hearing with aids)	0 (0)	0 (0)	n/a	0 (0)	n/a
Degenerative disc disease (e.g., spinal stenosis)	3 (5)	0 (0)	0.08	2 (3)	0.32
FCI Score, median (IQR)***	2 (1–3)	1 (0–1)	<0.01	1 (1–2)	<0.01

*Abbreviations*: ARDS, acute respiratory distress syndrome; COPD, chronic obstructive pulmonary disease; GI, gastrointestinal; MS, multiple sclerosis; TIA, transient ischemic attack.

* p-value comparing prospective data collection to chart abstraction, using McNemar’s test.

** p-value comparing retrospective data collection to chart abstraction, using McNemar’s test.

*** For the FCI score, comparison made using Wilcoxon signed-rank test.

There were no statistically significant differences in the prevalences of individual FCI comorbidities obtained by the retrospective collection from hospital discharge summaries compared to the gold standard. However, the median (IQR) FCI score calculated from hospital discharge summaries was significantly lower than from the gold standard (1 [1–2] vs. 2 [1–3], p < 0.01).

As compared to retrospective collection from discharge summaries, the prospective collection from hospital admission records detected 11 of 17 comorbidities significantly less frequently (Table 3). Myocardial infarction was the only comorbidity detected significantly more frequently from hospital admission records. The median (IQR) FCI score calculated from hospital admission records was significantly lower than that obtained from the discharge summaries (1 [0–2] vs. 2 [1–3], p < 0.01).

**Table 3:** Prevalence of Individual FCI Comorbidities by Data Collection Method (n = 421)

**FCI Comorbidity, n (%)**	**Prospective: Admission Records**	**Retrospective: Discharge Summaries**	p-value*
Arthritis (rheumatoid and osteoarthritis)	4 (1)	23 (5)	<0.01
Osteoporosis	4 (1)	10 (2)	0.03
Asthma	16 (4)	28 (7)	<0.01
COPD, emphysema, or ARDS	21 (5)	56 (13)	<0.01
Angina	1 (1)	1 (1)	1.00
Congestive heart failure (or heart disease)	80 (19)	84 (20)	0.07
Myocardial infarction	24 (6)	13 (3)	0.03
Neurological disease (e.g., MS or Parkinson’s)	7 (2)	55 (13)	<0.01
Stroke or TIA	34 (8)	25 (6)	0.32
Peripheral vascular disease	13 (3)	25 (6)	<0.01
Diabetes type I or II	105 (25)	81 (19)	0.74
Upper GI disease (e.g., ulcer, hernia, reflux)	26 (6)	77 (18)	<0.01
Depression	39 (9)	62 (15)	<0.01
Anxiety or panic disorders	7 (2)	22 (5)	<0.01
Visual impairment (e.g., cataracts, glaucoma)	2 (1)	9 (2)	<0.01
Hearing impairment (e.g., hard of hearing with aids)	1 (1)	1 (1)	0.32
Degenerative disc disease (e.g., spinal stenosis)	8 (2)	14 (3)	0.01
FCI Score, median (IQR)**	1 (0–2)	2 (1–3)	<0.01

*Abbreviations*: ARDS, acute respiratory distress syndrome; COPD, chronic obstructive pulmonary disease; GI, gastrointestinal; MS, multiple sclerosis; TIA, transient ischemic attack.

* Comparison made using McNemar’s test.

** For the FCI score, comparison made using Wilcoxon signed-rank test.

### Accuracy of using hospital admission records and discharge summaries for FCI

The sensitivity, specificity, positive and negative predictive values for detecting each of the individual FCI comorbidities by either the hospital admission records or hospital discharge summaries are presented in Table 4. Specifically, discharge summaries were more sensitive than the hospital admission records in detecting 9 of 17 comorbidities, including chronic respiratory (e.g., asthma, COPD), neurological, and psychiatric (e.g., depression, anxiety) disorders.

**Table 4:** Accuracy of Individual FCI Comorbidities by Data Collection Method Compared to Complete Chart Review (n = 59)

**FCI Comorbidity**	**Prospective:**				**Retrospective:**			
	**Admission Records**				**Discharge Summaries**			
	**Sn**	**Sp**	**PPV**	**NPV**	**Sn**	**Sp**	**PPV**	**NPV**
Arthritis (rheumatoid and osteoarthritis)	14%	88%	25%	78%	14%	8%	4%	25%
Osteoporosis	n/a	n/a	n/a	n/a	0%	23%	0%	75%
Asthma	22%	52%	13%	68%	67%	24%	21%	70%
COPD, emphysema, or ARDS	0%	67%	0%	84%	63%	22%	9%	82%
Angina	0%	50%	0%	50%	0%	50%	0%	50%
Congestive heart failure (or heart disease)	50%	19%	4%	86%	67%	26%	9%	88%
Myocardial infarction	50%	21%	4%	86%	50%	59%	8%	94%
Neurological disease (e.g., MS or Parkinson’s)	0%	89%	0%	91%	100%	23%	11%	100%
Stroke or TIA	100%	17%	15%	100%	80%	40%	16%	93%
Peripheral vascular disease	25%	63%	8%	87%	75%	31%	12%	91%
Diabetes type I or II	50%	27%	7%	83%	50%	40%	9%	88%
Upper GI disease (e.g., ulcer, hernia, reflux)	8%	75%	8%	75%	80%	39%	26%	88%
Depression	17%	53%	8%	74%	56%	33%	16%	76%
Anxiety or panic disorders	0%	74%	0%	77%	67%	30%	17%	80%
Visual impairment (e.g., cataracts, glaucoma)	n/a*	n/a	n/a	n/a	0%	36%	0%	83%
Hearing impairment (e.g., hard of hearing with aids)	n/a	n/a	n/a	n/a	n/a	n/a	n/a	n/a
Degenerative disc disease (e.g., spinal stenosis)	0%	60%	0%	71%	60%	45%	21%	82%

*Abbreviations*: ARDS, acute respiratory distress syndrome; BMI, body mass index; COPD, chronic obstructive pulmonary disease; GI, gastrointestinal; MS, multiple sclerosis; NPV, negative predictive value; PPV, positive predictive value; Sn, sensitivity; Sp, specificity; TIA, transient ischemic attack.

* n/a if there were no instances of this comorbidity to calculate Sn, Sp, PPV, or NPV.

### Predictive validity of the FCI score and SF-36 PFS at 1 year

The bivariate and multivariable linear regression results evaluating the association between the FCI score obtained from the three different methods of data collection and the SF-36 PFS score at 1 year are presented in Table 5. FCI scores calculated from retrospective data collection using discharge summaries explained slightly more variance in SF-36 PFS scores at 1 year than either prospective data collection or complete chart review data, with R^2^ for the multivariable model of 0.20, 0.18, and 0.17, respectively.

**Table 5:** Association Between FCI Score and SF-36 PFS at 1 Year (n = 47)*

	**Bivariate**	Multivariate**	**R-squared for Multivariate**
	**Coefficient (95% CI)**	**Coefficient (95% CI)**	
Prospective (ICAP original)	−6.1 (−10.8 to −1.3)	−3.7 (−8.2 to 0.8)	0.17
Retrospective (Discharge summary)	−6.0 (−9.4 to −2.7)	−4.5 (−7.7 to −1.3)	0.20
Chart review (Gold standard)	−6.9 (−12.4 to −1.5)	−3.4 (−7.3 to 0.5)	0.18

*Abbreviations*: CI, confidence interval; RR, relative risk; SF-36 PFS, Short Form-36 Physical Function Subscale.

* Linear regression analysis with 47 patients that had complete chart review and completed SF-36 at 1 year. Reported coefficients represent the change in SF-36 PFS score at 1 year for every 1 unit increase in FCI score. A 1.5-unit difference in FCI score corresponds to a minimum important difference in SF-36 PFS score at 1 year.

** Adjusted for age and sex at ICU admission.

Multivariate models, adjusted for age and gender, using the CCI or the chronic health points comorbidity measure from the APACHE II score explained less variance in SF-36 PFS scores at 1 year than the FCI score from hospital discharge summaries, with R^2^ for the multivariate model of 0.17 and 0.16, respectively.

## Discussion

In our study of 421 acute lung injury patients, we found substantial inter-rater reliability for FCI across trained data collectors. A number of comorbidities were underdetected by the prospectively collecting data using hospital admission records versus a complete chart review. There were no significant differences in the prevalence of comorbidities detected retrospectively using discharge summaries versus complete chart review. Both the prospective and retrospective data collection methods significantly underestimated the overall FCI score compared to the gold standard method. Using multivariable regression, adjusting for age and gender, FCI scores obtained from discharge summaries explained more variance in SF-36 PFS score at 1 year than FCI score obtained from hospital admission records or complete chart review, but all three methods had similar magnitudes of association with PFS, suggesting comparative predictive value. Moreover, FCI scores explained more variance in SF-36 PFS at 1 year than the Charlson comorbidity index or the chronic health points of the APACHE II score at ICU admission.

Adjustment for patients’ baseline comorbidities is essential to understand the independent contributions of various exposures or therapies to long-term physical function and outcomes in clinical research. To the best of our knowledge, this study is the first to evaluate inter-rater reliability of FCI. Furthermore, despite both hospital admission records and discharge summaries underestimating the FCI score (as compared to the gold standard of complete chart abstraction), all three methods demonstrated similar associations with the SF-36 PFS at 1 year, suggesting comparative predictive value for this outcome. Given that the time and effort required to collect comorbidity information from a discharge summary is much more efficient than complete chart review, the use of retrospective data collection from hospital discharge summaries is a reliable and valid option for clinical research that requires comorbidity information for predicting SF-36 PFS at 1 year.

Ideally, the comorbidity information obtained from a variety of patient documentation sources should be similar, although there are likely important reasons why different data acquisition methods may vary in detection of individual comorbidities. Specifically in our study, due to the unpredictable nature of critical illness, patients admitted to ICU may have a more complete history deferred, including a review of comorbidity information (e.g., arthritis, osteoporosis, depression) unrelated to the acute illness, as the health care team initially tries to resuscitate and stabilize the patient. Thus, prospective data collection using patient records shortly after ICU admission may underdetect certain comorbidity information. Conversely, discharge summaries have the opportunity to codify the comorbidity information documented by a number of health care providers over the course of an entire hospital admission, allowing for potentially more complete or more important comorbidity to be captured.

Given the variability in the sensitivity of detecting various classes of comorbidities by data collection method, the optimal choice for data collection may vary based on the population studied, the outcome of interest, and question to be answered. This is important since prevalent comorbidities which may go undetected could result in substantial bias in the FCI score which is not accounted for [8]. Conversely, prospective data collection of comorbidity information may be preferred if study coordinators are already collecting other data from the medical chart at admission, and the population being studied is expected to have few pre-existing comorbid illnesses (e.g., younger patients).

The FCI is a novel and unique comorbidity scale developed with physical function as the primary outcome. In creating the FCI, the investigators hypothesized that diagnoses associated physical function would be different than those associated with mortality as used in the popular CCI [9-13]. Thus, FCI should outperform indices designed with mortality as the outcome of interest (e.g., CCI) in predicting physical function. Our results are consistent with a previous study demonstrating that, comparing FCI to the CCI and Kaplan-Feinstein index (KFI), the FCI accounted for more variation in the SF-36 PFS, highlighting the importance of using risk models designed to predict a specific outcome.

Our study has potential limitations. While we did not explicitly evaluate the time required to collect comorbidity information by each of the three methods, it was clear that retrospective data collection using electronic discharge summaries was much less time consuming that complete chart review, and more efficient than prospective data collection from admission records, especially since it could be completed remotely using computer access to the records. Furthermore, we did not evaluate whether a hybrid method (e.g., combined prospective and retrospective data collection) would be superior to either method alone. However, the goal of our study was to demonstrate whether a more efficient method of data collection (i.e., using electronic discharge summaries as a retrospective form of data collection) would have acceptable inter-rater reliability and sufficient validity compared to the gold standard (complete chart review). Indeed, since we did collect comorbidity data both prospectively and retrospectively, the final comorbidity dataset for the ICAP parent study included comorbidities from both methods. Finally, our results were obtained from a population of ALI patients, and as such, may not be generalizable to other populations. However, the FCI has been validated in ALI patients [10], and given our hypothesis that more detailed and comprehensive comorbidity information may be collected and documented in the medical record over the course of an entire hospital admission, our results are likely to be applicable to other groups of hospitalized patients.

## Conclusions

FCI comorbidity information collected retrospectively from discharge summaries had excellent inter-rater reliability when performed by trained data collectors. Retrospective data collection using electronic hospital dis3charge summaries represents a reliable, and valid method for collecting FCI comorbidity information. With the increasing availability of electronic hospital discharge summaries, the use of retrospective data collection for FCI comorbidity information is an important tool for ICU outcomes research, and potentially for clinical research in other areas of health care.

## Competing interests

The authors declare that they have no competing interests.

## Author’ contributions

Study concept and design: EF, PJP, DMN, Acquisition of data: EF, JMG, SC, Analysis and interpretation of data: EF, EC, DMN, Drafting of the manuscript: EF, DMN, Critical revision of the manuscript for important intellectual content: EF; JMG; SC; EC; PJP; DMN, Statistical analysis: EF, EC, Study supervision: EF, PJP, DMN, All authors read and approved the final manuscript.

## Funding and role of sponsor

This research is supported by the National Institutes of Health (Acute Lung Injury SCCOR grant P050 HL 73994). EF is supported by a Fellowship Award from the Canadian Institutes of Health Research. The funding bodies had no role in the design and conduct of the study; collection, management, analysis, and interpretation of the data; and preparation, review, or approval of the manuscript.

## Pre-publication history

The pre-publication history for this paper can be accessed here:

http://www.biomedcentral.com/1471-2253/12/21/prepub
